# Intracytoplasmic inclusions in chronic lymphocytic leukemia cells of an elderly person living with human immunodeficiency virus and presenting with Richter transformation to Hodgkin lymphoma

**DOI:** 10.1002/jha2.1087

**Published:** 2025-01-10

**Authors:** Evashin Pillay, Ayanda Gugulethu Precious Jali, Nadine Rapiti

**Affiliations:** ^1^ University of KwaZulu‐Natal & National Health Laboratory Service Inkosi Albert Luthuli Central & King Edward VIII Hospitals Durban South Africa; ^2^ University of KwaZulu‐Natal & KwaZulu‐Natal Provincial Department of Health Inkosi Albert Luthuli Central & King Edward VIII Hospitals Durban South Africa

1

A 65‐year‐old South African man living with HIV (human immunodeficiency virus; CD4 count: 557 cells/µL; HIV viral load: undetectable) since 2008 (medication: fixed‐dose combination antiretroviral therapy—tenofovir, lamivudine, dolutegravir) presented with a progressively enlarging left‐sided neck mass of 9 months’ duration. His associated symptoms included dysphagia and significant weight loss (> 10% body weight during the previous 6 months).

At the initial consultation, his general examination was remarkable for muscle wasting, pallor, and generalized significant lymphadenopathy (LAD), most notably as a bulky left‐sided neck mass (> 10 cm). Systemic examination revealed tracheal deviation to the right, reduced breath sounds in the upper zone of the left lung, and epigastric fullness; no hepatosplenomegaly, ascites, or other pertinent signs. The overall clinical impression was that of an elderly, HIV‐positive man presenting with features of bulky lymphomatous disease complicated by esophageal and tracheal obstruction.

A full blood count revealed a marked leukocytosis (white blood cells 73.85 × 10^9^/L; lymphocytes 92% − 67.94 × 10^9^/L; neutrophils 6% − 4.43 × 10^9^/L), mild anemia (hemoglobin 11.9 g/dL, range 13.0−17.0; hematocrit 0.383 L/L), and mild thrombocytopenia (platelets 159 × 10^9^/L, range 171−388); corrected reticulocyte count 0.92%.

Examination of the peripheral blood smear confirmed the lymphocytosis and revealed mainly small mature lymphocytes, in addition to a subpopulation (8%) of medium‐sized, plasmacytoid counterparts (Figure 1A−I). The small tumor cells (Figure 1A,B; green arrows) and smudge cells (Figure 1H; top left corner) were morphologically characteristic of a mature lymphoid neoplasm. Nonetheless, a striking morphological feature was that of circumscribed, opaque, globular intracytoplasmic inclusions of variable size and number within the uniformly small lymphocytes (Figure 1A,B; black arrows) and the pleiomorphic medium‐sized lymphocytes (Figure 1C−I; red arrows).

**FIGURE 1:**
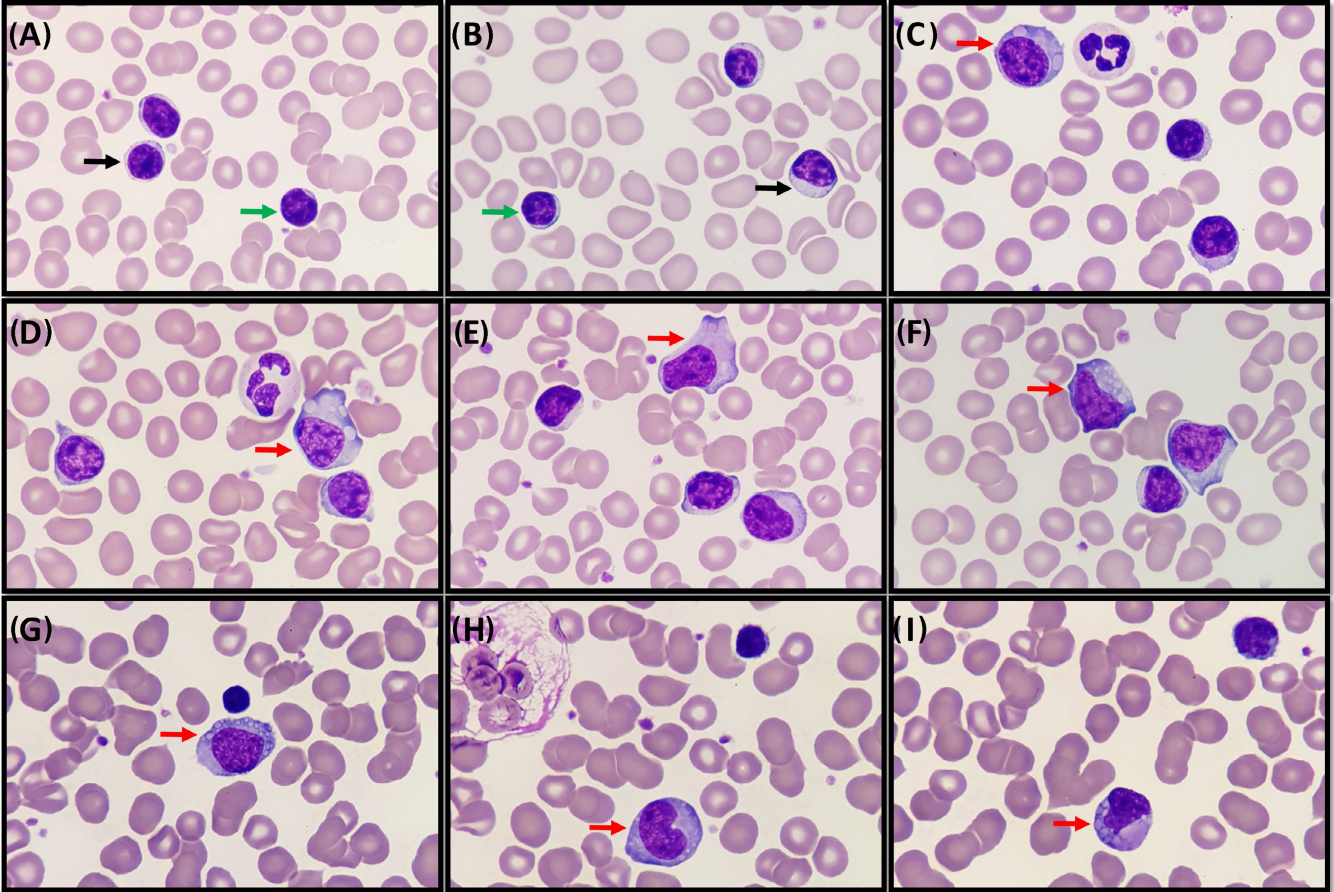
(A−I): Peripheral blood smear (May−Grünwald−Giemsa; 100 × objective). Intracytoplasmic inclusions within CLL lymphocytes: small tumor cells without inclusions (A, B; green arrows); smudge cell (H; top left corner); small lymphocytes (A, B; black arrows) and pleiomorphic medium‐sized lymphocytes (C−I; red arrows) with circumscribed, opaque, globular intracytoplasmic inclusions of variable size and number.

Peripheral blood immunophenotyping identified a weakly lambda‐restricted B‐cell population, positive for CD5, CD19, CD20 (weak), CD22 (weak), CD23, and CD200, without expression of FMC7 or CD10, diagnostic of chronic lymphocytic leukemia (CLL) (Matutes score 5) [1]—Rai stage 1 (modified risk status: intermediate) and Binet stage B [2].

Other relevant results: beta 2‐microglobulin (2.1 mg/L, range 0.6−2.4), protein electrophoresis (no paraprotein detected), serum free light chains (decreased Kappa:Lambda ratio of 0.25, range 0.26−1.65), and immunoglobulins (Ig) (normal IgG and IgA; reduced IgM).

A staging computed tomography scan demonstrated bulky LAD in the left cervical area extending from the submandibular to the supraclavicular region (9.5 × 11.2 × 13 cm—high tumor burden) [2], causing deviation of the oropharynx, larynx, and superior trachea to the right. Additionally, significant LAD (> 1 cm) was noted in the mediastinal (up to 3.6 cm), bilateral hilar (up to 1.7 cm), bilateral axillae (up to 3.2 cm), pre‐ and para‐aortic, para‐caval, and mesenteric (up to 3.1 cm) regions. No cardiopulmonary, abdominal, or pelvic pathologies were detected.

The staging bone marrow biopsy analyses by morphology and flow cytometry confirmed the diagnosis of CLL with > 40% clonal lymphocytes and no features of large cell transformation. The corresponding CLL FISH (fluorescence in situ hybridization) panel was positive for deletion 11q22 (unfavorable risk) [2] and conventional cytogenetics by CpG‐stimulated metaphase analysis did not reveal a complex karyotype.

Subsequently, an excisional lymph node biopsy was compatible with a histological diagnosis of CLL and EBER‐ISH (EBV‐encoded RNA in situ hybridization) positive classic Hodgkin lymphoma (cHL; lymphocyte‐rich subtype), that is, Hodgkin variant Richter transformation, for which there are currently no established, optimal standard of care treatment protocols [3]. As a suitable clinical trial was not available for enrollment, the patient was initiated on standard, primary systemic chemotherapy with ABVD (doxorubicin, bleomycin, vinblastine, dacarbazine). Post first‐line chemotherapy, an interim whole‐body positron emission tomography scan showed progressive disease in the involved lymph node groups; an assessment of primary refractory disease was concluded. The patient was subsequently counseled extensively and opted for salvage chemotherapy comprised of ICE (ifosfamide, carboplatin, etoposide), in addition to best supportive care in an outpatient setting.

The presence of intracytoplasmic inclusions within CLL lymphocytes is uncommon. Previous case reports mainly describe crystalline or tubular, “filamentous‐like” intracytoplasmic inclusions that are thought to represent an abnormal immunoglobulin product, the clinical and prognostic significance of which is uncertain [4]. However, their recognition should prompt further investigations to exclude a mature lymphoproliferative neoplasm.

Overall, this case highlights an exceptionally rare pathologic entity evidenced by variably sized, circumscribed, globular cytoplasmic inclusions within a spectrum of CLL cells of an elderly patient living with HIV. Furthermore, the clinical presentation of Richter transformation masquerading as bulky lymphoma disease reiterates the diagnostic, prognostic, and therapeutic value of an excisional lymph node biopsy in such cases.

## CONFLICT OF INTEREST STATEMENT

The authors declare that they have no conflict of interest.

## FUNDING INFORMATION

The authors received no specific funding for this work.

## ETHICS APPROVAL STATEMENT

Ethical approval was obtained from the Biomedical Research Ethics Committee affiliated with the University.

## PATIENT CONSENT STATEMENT

Permission was granted by the patient to publish clinical information and photographic material relating to their medical condition.

## CLINICAL TRIAL REGISTRATION (INCLUDING TRIAL NUMBER)

The authors have confirmed clinical trial registration is not needed for this submission.

## Data Availability

The data that support the findings of this study are available from the corresponding author upon reasonable request.
